# Comparison of radiomics-based models for detection of Modic type 1 changes in photon-counting detector CT images of the lumbar spine

**DOI:** 10.1007/s00256-026-05247-7

**Published:** 2026-05-08

**Authors:** Adrian A. Marth, Benjamin Fritz, Reto Sutter

**Affiliations:** 1 Department of Radiology, Balgrist University Hospital, Zurich, Switzerland; 2 Medical Faculty, University of Zurich, Zurich, Switzerland; 3 Department of Radiology and Biomedical Imaging, University of California, San Francisco, CA USA

**Keywords:** Lumbar spine, Modic change, Photon-counting detector CT, Radiomics, Machine learning

## Abstract

**Objective:**

To compare diagnostic performance of four radiomics-based machine learning models for detecting Modic type 1-changes of the lumbar spine in photon-counting detector (PCD)-CT images, using MRI as the reference standard.

**Materials and methods:**

In this retrospective single-center study, 60 patients who underwent lumbar spine PCD-CT and MRI within a one-week interval showing Modic type 1-changes were analyzed. A total of 105 radiomic features were extracted from 360 segmented vertebrae, of which 348 were included in the final analysis after quality control. Least Absolute Shrinkage and Selection Operator (LASSO), Random Forest, Extreme Gradient Boosting (XGBoost), and support vector machines (SVM) were trained and evaluated using nested cross-validation. Discriminatory performance of the models was evaluated by area under the receiver operating characteristic curve (AUC). AUC values were compared using the DeLong Test with Benjamini–Hochberg correction to adjust for multiple testing. Diagnostic accuracy was assessed by calculating sensitivity, specificity and F1-score for each model.

**Results:**

LASSO achieved the highest AUC (0.842, 95% CI 0.793—0.891), pairwise comparisons did not show significant differences across the models (*p* ≥ 0.337). Sensitivity was highest for LASSO (0.756, 95% CI 0.662—0.846), whereas specificity was highest for SVM (0.929, 95% CI 0.896—0.958). The highest F1-score was observed for LASSO (0.605, 95% CI 0.521—0.679).

**Conclusion:**

Four radiomics-based machine learning models demonstrated similar high discriminatory performance but differing diagnostic accuracy for detecting Modic type 1-changes on PCD-CT images. These results support the feasibility of radiomics for evaluation of pathologies beyond visual inspection, although further validation is required to determine clinical applicability.

## Introduction

Endplate signal changes are commonly visualized on MR images of the lumbar spine, and were first described by Modic et al. in 1988 [1]. These changes were classified into three subtypes according to their bone marrow signal patterns: Type 1 changes (MC-1) are defined by the presence of bone marrow edema-like lesions and were described as hypointense on T1-weighted and hyperintense on T2-weighted images, Type 2 changes (MC-2) contain fatty marrow and are hyperintense on T1-weighted and hyperintense or isointense on T2-weighted images, and Type 3 changes (MC-3) are hypointense on both T1- and T2-weighted sequences [1, 2].

Modic changes are more prevalent among individuals with low back pain, with a pooled prevalence of approximately 43% in symptomatic individuals, compared with approximately 6% in asymptomatic populations [3]. Among the different subtypes, MC-1 show the strongest association with low back pain [4]. Moreover, Modic changes are associated with disc degeneration and/or herniation [5, 6], and seem to play an important role in fusion rates after spinal interbody fusion [7]. Histomorphometric studies of vertebral biopsies have highlighted the dynamic interaction between bone and marrow compartments in Modic changes, showing increased bone turnover in MC-1, diminished bone formation in MC-2, and sclerotic bone changes in MC-3 [8].

While CT has been used by a few authors to detect sclerotic Modic changes [7, 9] or to differentiate Modic subtypes based on Hounsfield units [10], its value to detect MC-1 changes remains very limited.

Radiomics has emerged as an expanding application of computer-aided medical image analysis, leveraging the vast amount of quantitative information embedded in cross-sectional images through the spatial distribution and gray-level intensities of pixels, which may capture imaging patterns beyond visual assessment [11]. The improved spatial resolution, lower electronic noise, higher contrast-to-noise ratio, and the potential for inherent energy-resolved imaging of photon-counting detector (PCD)-CT scanners may facilitate broader application of radiomics in medical image analysis, as early evidence suggests improvements in radiomics feature stability [12, 13]. However, no study has yet specifically investigated the potential of radiomics to detect MC-1 changes on CT images, and particularly not using PCD-CT.

Therefore, this study aimed to compare different machine learning models for feature-based detection of MC-1 changes of the lumbar spine vertebrae in PCD-CT images, using MRI as the reference standard.

## Materials and methods

### Patient characteristics

**Fig. 1:**
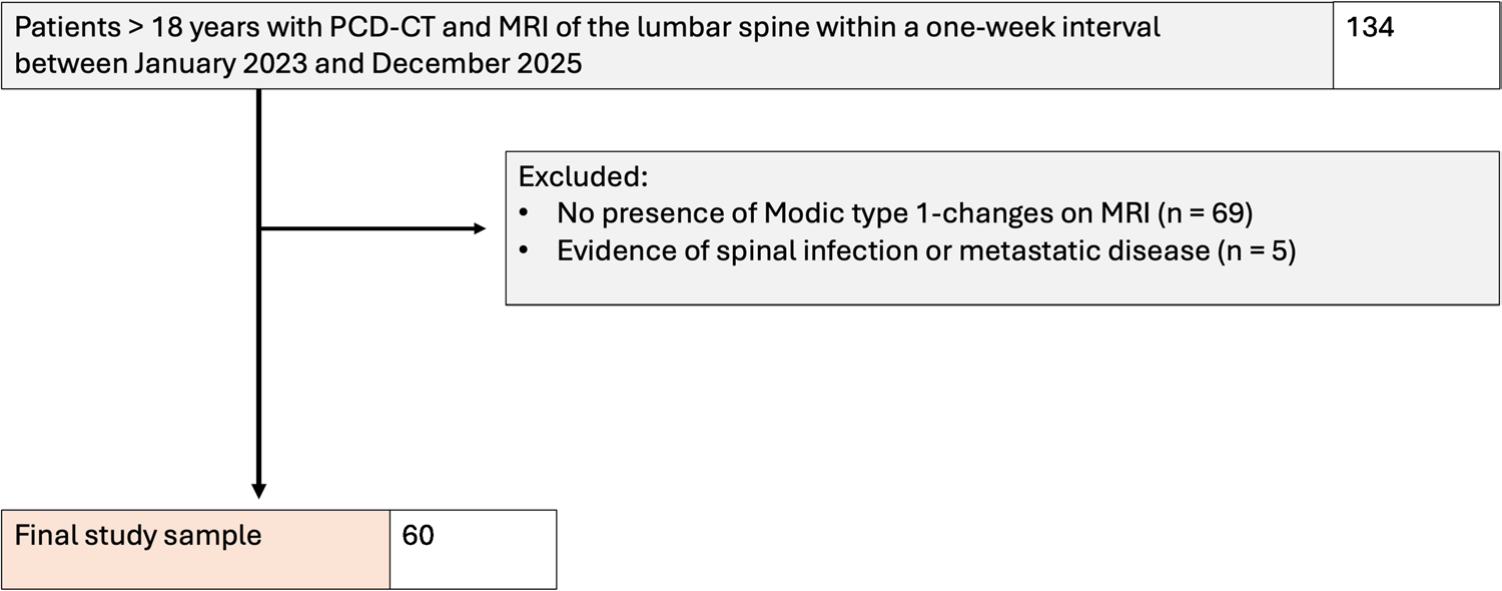
Patient selection process. *PCD-CT Photon-counting detector CT*

This retrospective single-center study was approved by the local ethics committee, and the requirement for informed consent was waived. Patients > 18 years who had undergone both PCD-CT and lumbar spine MRI within a one-week interval between January 2023 and December 2025 were consecutively identified through the institutional Picture Archiving and Communications System (PACS). Inclusion criteria were presence of MC-1 changes between L1 and S1 on MRI, and no evidence of spinal infection or metastatic disease on MRI and/or CT images. After applying these criteria, 60 patients were included in the final cohort. The patient selection process is illustrated in Fig. 1.

### Imaging protocol and evaluation

PCD-CT acquisition and reconstruction parameters are summarized in Table 1, while MRI acquisition parameters are provided in Appendix 1. In patients with metal implants, a dedicated metal artifact reduction protocol was used for MR examinations, whereas PCD-CT protocols were uniform across the whole cohort. Image evaluation was performed by a fellowship-trained musculoskeletal radiologist (AAM).

**Table 1:** Photon counting detector CT acquisition parameters for CT of the lumbar spine, and reconstruction parameters for sagittal CT images of the lumbar spine

Tube potential (kVp)	140
Collimation (mm)	144 × 0.4
Pitch factor	0.8
Rotation time (s)	1.0
Focal spot size (mm)	0.8 × 1.2
Tin prefiltration	Yes
Reconstruction kernel	Br64
Slice thickness (mm)	3
Slice increment (mm)	3
Matrix size	512 × 512
Pixel spacing (mm)	0.38 × 0.38

*kVp* kilovolt peak

MC-1 changes were assessed on a per-vertebral body level on sagittal T1-weighted and T2-weighted MR images according to the original classification [1] in conjunction with the sagittal short tau inversion recovery sequences, and scored as present (positive) or absent (negative). A vertebra was classified as positive if MC-1 changes were present at either the superior or inferior endplate. Borderline cases were resolved by requiring signal changes consistent with MC-1 on all available sequences, i.e. hypointense on T1-weighted images and hyperintense on T2-weighted/short tau inversion recovery images. Vertebrae without definitive MC-1 features on all sequences were classified as negative. 

### Image segmentation

**Fig. 2:**
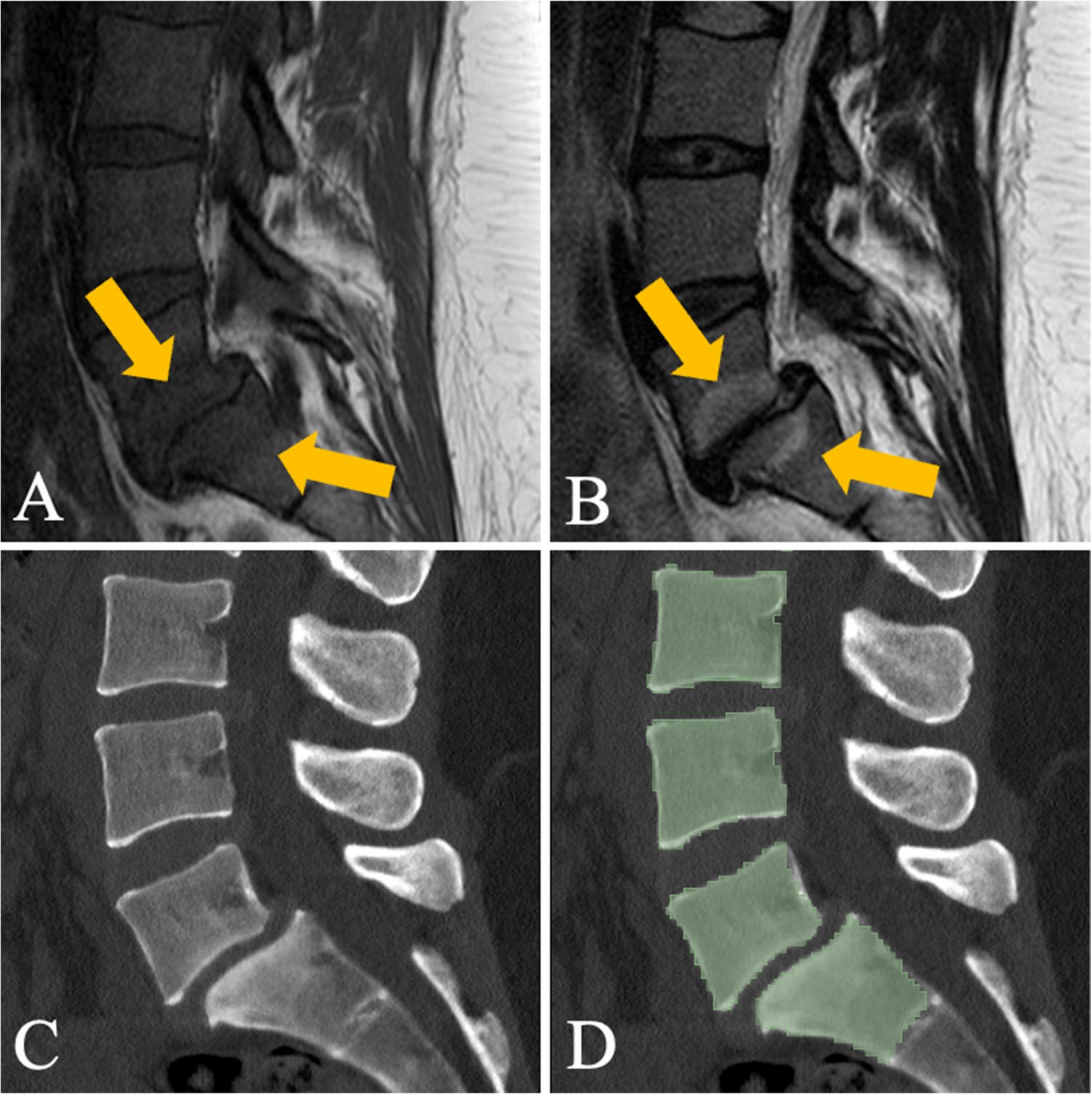
Representative sagittal T1-weighted (**a**) and T2-weighted MR image (**b**) of the lumbar spine of a 35-year-old female patient with bilateral spondylolysis and Modic type 1 changes of the L5/S1 segment (arrows). The subsequent photon-counting detector CT examination performed 5 days later is shown in (**c**). Overlaid masks of the vertebral bodies of L3-S1 for radiomics feature extraction are shown in (**d**). For this patient, the LASSO model estimated probabilities of Modic type 1 changes of 0.06 at L3, 0.08 at L4, 0.91 at L5, and 0.88 at S1

**Fig. 3:**
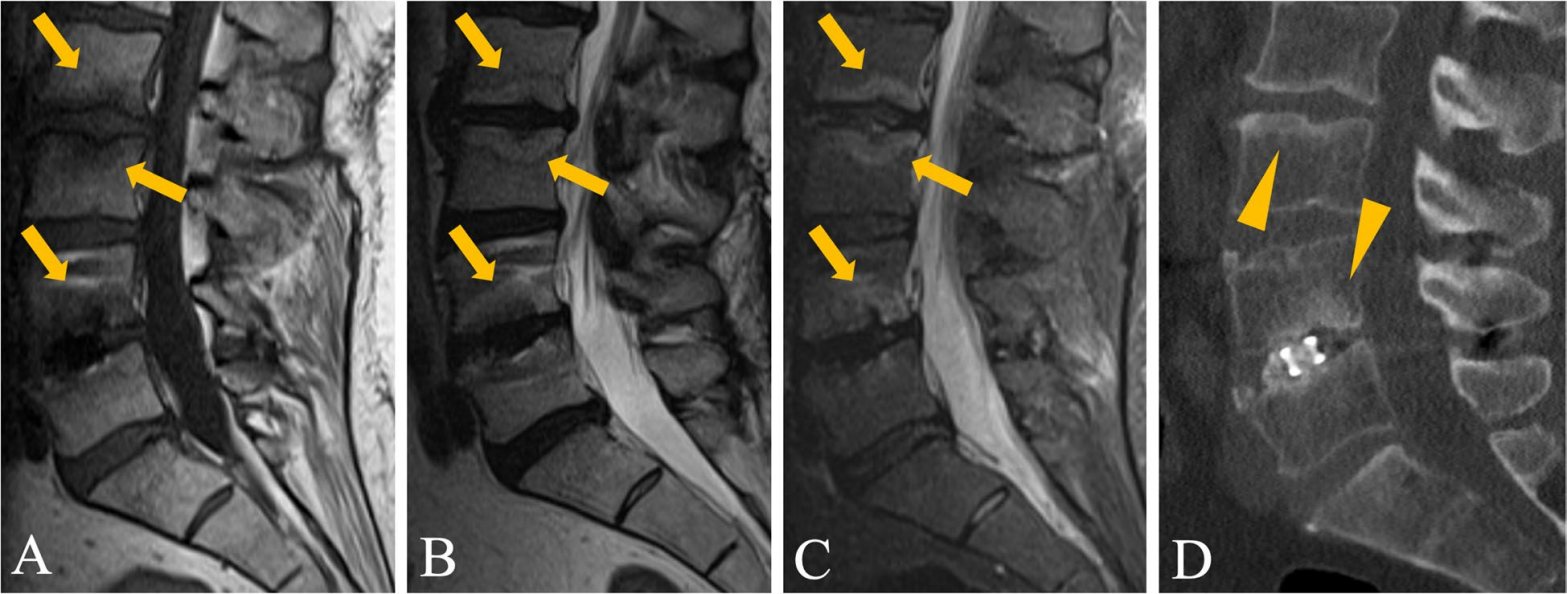
Sagittal T1-weighted images (**a**) and T2-weighted images (**b**) of a 57-year-old female patient, who underwent L4/5 interbody fusion and cage implantation. Arrows indicate the Modic type 1 changes of the endplates, along with bone marrow edema-like lesions in the fluid-sensitive short tau inversion recovery image (**c**). The subsequent photon-counting detector CT examination (**d**) revealed minor sclerosis along the vertebral endplates of L3 and L4 (arrowheads). The LASSO model estimated probabilities of MC-1 changes of 0.92 at L2, 0.90 at L3, 0.85 at L4, 0.24 at L5, and 0.05 at S1

Lumbar vertebral bodies were segmented automatically on sagittal PCD-CT reconstructions using the deep learning-based tool TotalSegmentator (v2.5.0) by Wasserthal et al. [14], which has a high reproducibility with reported mean Dice similarity coefficients of 0.94 for this task [14]. Labeling of vertebral bodies was performed post-hoc using 3D Slicer (v5.8.1, [15]) followed by manual quality control for segmentation errors. Vertebrae with segmentation errors or incomplete visualization were excluded during this step prior to radiomics feature extraction and model training. Representative images of patients with MC-1 changes in MRI and corresponding PCD-CT are shown in Figs. 2 and 3.

### Radiomics feature extraction

A total of 105 radiomics features were extracted using SimpleITK [16] and PyRadiomics [17]. For each vertebra, the source CT was read as a three-dimensional DICOM series and the corresponding vertebral mask was loaded and binarized. Masks were resampled to the native CT image geometry with nearest-neighbor interpolation to ensure voxel-wise correspondence. No intensity resampling or voxel-size normalization was applied. The extractor was configured to enable all PyRadiomics feature classes, resulting in the calculation of first-order statistics (*n* = 18), three-dimensional shape features (*n* = 15), and multiple texture feature families (*n* = 72), including gray-level co-occurrence matrix (GLCM), gray-level run-length matrix (GLRLM), gray-level size-zone matrix (GLSZM), gray-level dependence matrix (GLDM), and neighboring gray-tone difference matrix (NGTDM). Gray-level discretization employed a fixed bin count of 32 within the region of interest intensity range, and no bin width was applied. A geometry tolerance of one voxel was used for mesh- and shape-based computations. After feature extraction, outputs were restricted to numeric and finite values only.

Gray-level discretization was performed using a fixed bin count of 32 within the region-of-interest intensity range to ensure consistent feature extraction across all cases. Intensity normalization was not applied, as CT attenuation values are inherently standardized in Hounsfield units, enabling direct comparability across patients. Likewise, voxel-size normalization was not performed, as all images were acquired using a uniform acquisition and reconstruction protocol with consistent spatial resolution. A geometry tolerance of one voxel was used for mesh- and shape-based computations.

### Radiomics modeling

Radiomics modeling was conducted in R (v4.5.0, R Foundation for Statistical Computing, Vienna, Austria). Four supervised learning algorithms were compared: Least Absolute Shrinkage and Selection Operator (LASSO) logistic regression, Random Forest, Extreme Gradient Boosting (XGBoost), and Support Vector Machines with radial basis kernel (SVM). Class imbalance was addressed by inverse-frequency weighting, applied as per-observation weights in LASSO, class-level weights in Random Forest, increased weighting of positive cases in XGBoost, and class-specific weighting in SVM. Prior to training, all features were standardized by centering and scaling.

### Validation and feature selection

Model performance was assessed using a nested cross-validation framework. The outer loop comprised five stratified folds to preserve class balance. Within each outer training set, recursive feature elimination was performed using an inner five-fold cross-validation. Folds were constructed at the patient level to ensure that all vertebrae from a given patient were assigned to the same fold. Candidate feature subset sizes ranged from 5 to 20 in increments of 5. Hyperparameters were optimized in the inner loop by grid search, using the area under the receiver operating characteristic curve (AUC) as selection metric. This process was repeated across all outer folds, and pooled out-of-sample predictions were retained for subsequent analysis. The unit of analysis was the vertebral body; to account for within-patient clustering, folds were constructed at the patient level, ensuring that all vertebrae from a given patient were assigned exclusively to either training or test sets.

### Model performance

Model performance was quantified using the AUC with 95% confidence intervals (CIs) derived by 2,000 stratified bootstrap resamples overall and on a per-vertebra basis (L1-S1). CIs were derived from pooled out-of-sample predictions and did not explicitly account for intra-patient clustering. In addition, sensitivity, specificity, precision (positive predictive value), and the F1 score (harmonic mean of precision and sensitivity) were calculated. Calibration was assessed using the Brier score (mean squared error between predicted probabilities and observed outcomes). Calibration curves were derived from the pooled out-of-sample predictions obtained from the outer folds of the nested cross-validation, and bootstrap optimism correction was applied to these pooled predictions to account for potential overfitting.

### Statistical analysis

Statistical analyses were performed using R (v4.5.0, R Foundation for Statistical Computing, Vienna, Austria). Pairwise comparisons of AUC between models were conducted using the DeLong test. To account for multiple pairwise comparisons, we controlled the false discovery rate at 5% using the Benjamini-Hochberg procedure. Statistical significance was set at *p* < 0.05 after correction.

### Feature importance

Feature importance was derived separately for each model: For LASSO, non-zero regression coefficients at the optimal penalty parameter were extracted, for Random Forest, importance was quantified by mean decrease in accuracy, while for XGBoost, importance was computed by the “gain” metric from the final model. Since SVMs do not yield interpretable feature importance, no direct importance measures were reported.

## Results

### Patient characteristics

The study cohort comprised 60 patients (men, 34/60 [56.7%]; women, 26/60 [43.3%]) with a mean age of 64.0 ± 16.1 years (range, 19.4–84.4 years). Across all 360 vertebrae (L1–S1), MC-1 changes were prevalent in 82/360 cases (22.8%). Prevalence of MC-1 changes by vertebral were as follows: L1 *n* = 5 (8.3%), L2 *n* = 13 (21.7%), L3 *n* = 9 (15.0%), L4 *n* = 14 (23.3%), L5 *n* = 23 (38.3%), and S1 *n* = 18 (30.0%). Metallic implants were present in 35/60 patients (58.3%).

### Model performance and calibration

**Fig. 4:**
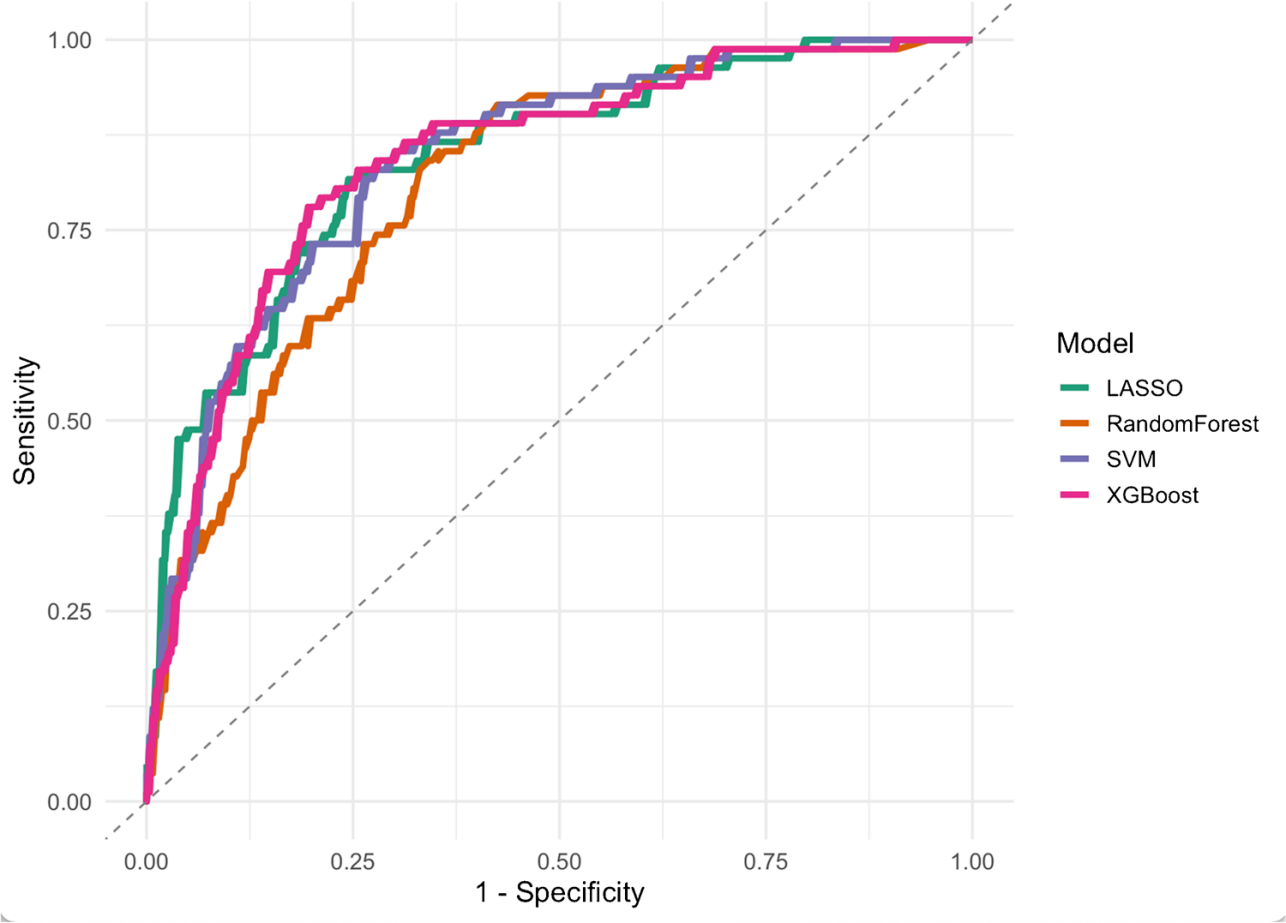
Receiver operating characteristic (ROC) curves derived from pooled predictions from nested cross-validation. Area under the curve, given with 95% Confidence Interval in parentheses was 0.842 (0.793–0.891) for LASSO, 0.841 (0.793–0.890) for XGBoost, 0.841 (0.794–0.888) for SVM, and 0.811 (0.761–0.860) for Random Forest. *LASSO, Least Absolute Shrinkage and Selection Operator; XGBoost, Extreme Gradient Boosting; SVM, Support Vector Machine*

**Fig. 5:**
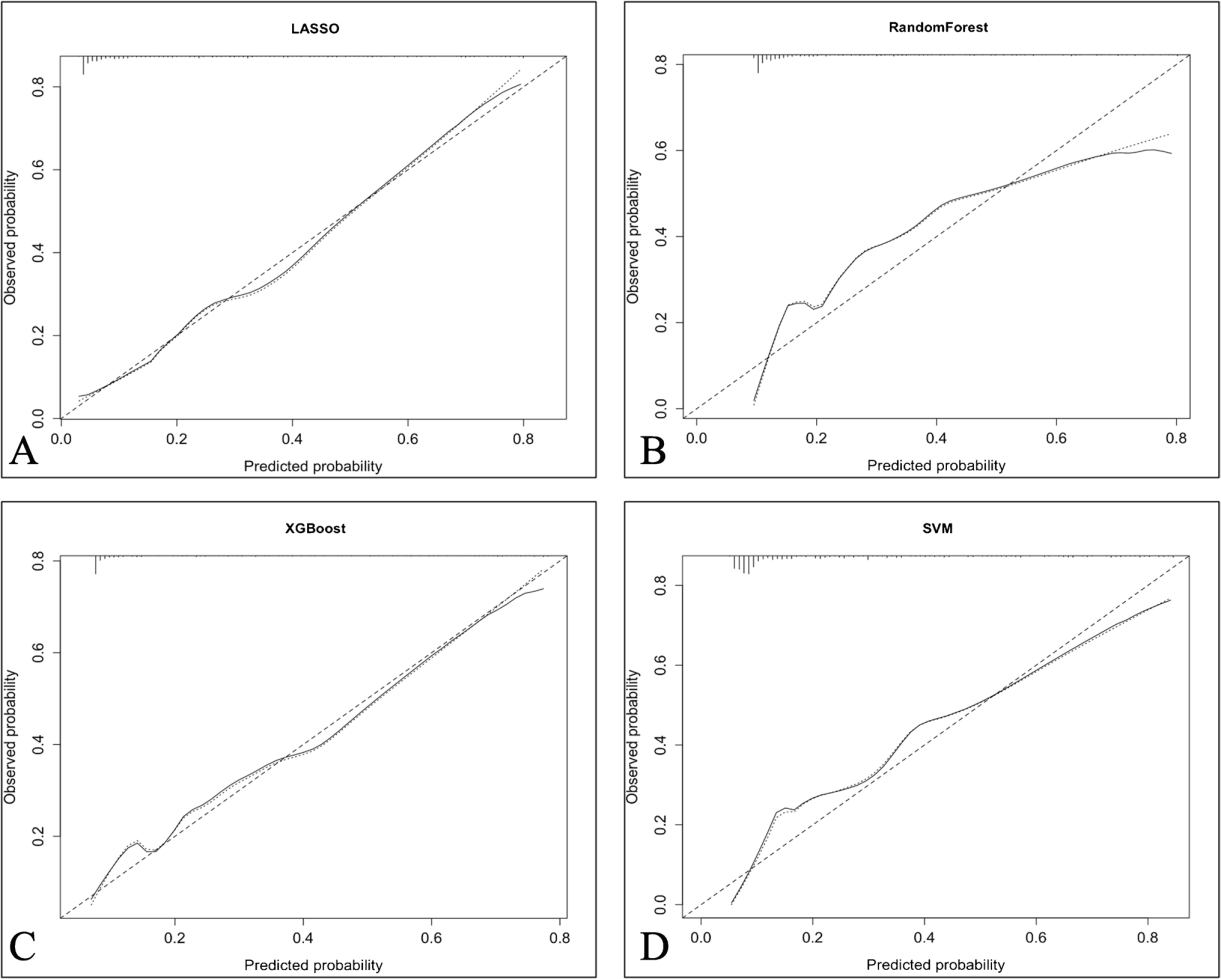
Bias-corrected calibration curves (solid lines) and apparent, uncorrected curves (dashed lines) are shown for LASSO (**a**), Random Forest (**b**), XGBoost (**c**), and SVM (**d**). The straight diagonal line indicates perfect calibration. Curves were obtained using logistic calibration with bootstrap optimism correction. The x-axis represents predicted probability, while the y-axis represents observed probability. Short tick marks along the axes indicate the distribution of predicted risks. *LASSO, Least Absolute Shrinkage and Selection Operator; XGBoost, Extreme Gradient Boosting; SVM, Support Vector Machine*

Following manual quality control, 7 vertebrae with segmentation errors and 5 vertebrae with incomplete visualization were excluded, yielding a final dataset of 348 lumbar vertebrae for further analysis. LASSO achieved the highest AUC (0.842, 95% CI 0.793–0.891), followed by XGBoost (0.841, 95% CI 0.793–0.890), SVM (0.841, 95% CI 0.794–0.888), and Random Forest (0.811, 95% CI 0.761–0.860). Receiver operating characteristic curves for all models are shown in Fig. 4. Pairwise comparisons revealed no significant differences in AUC between the models (*p* ≥ 0.337, Table 2). AUCs per vertebra for all models are shown in Table 3 and ranged from 0.722 (95% CI 0.399–1.000) for L1 (XGBoost) to 0.964 (95% CI 0.920–1.000) for L3 (SVM). Diagnostic performance of the models is summarized in Table 4: LASSO achieved the highest sensitivity (0.756, 95% CI: 0.662–0.846), while Random Forest achieved the lowest sensitivity (0.378, 95% CI: 0.275–0.482). Conversely, the highest specificity was achieved with SVM (0.929, 95% CI 0.896–0.958), and the lowest specificity was observed for LASSO (0.771, 95% CI 0.721–0.819). Precision ranged from 0.504 (95% CI 0.414–0.589) for LASSO to 0.672 (95% CI 0.547–0.789) for SVM. The highest F1 score was observed for LASSO (0.605, 95% CI 0.521–0.679). Brier scores ranged from 0.128 (95% CI 0.108–0.151) for SVM to 0.155 (95% CI 0.127–0.185) for XGBoost (Table 4). Calibration plots for the models are shown in Fig. 5.

**Table 2:** DeLong tests for the difference in area under the receiver operating characteristic curve (AUC) for different machine learning models. Unadjusted and Benjamini-Hochberg adjusted p-values to adjust for multiple testing are given, assuming a false discovery rate of 5%

Model 1	Model 2	Unadjusted p-value	Benjamini-Hochberg adjusted *p*-value
LASSO	Random Forest	0.144	0.337
LASSO	XGBoost	0.979	0.991
LASSO	SVM	0.966	0.991
Random Forest	XGBoost	0.169	0.337
Random Forest	SVM	0.091	0.337
XGBoost	SVM	0.991	0.991

*LASSO *Least absolute shrinkage and selection operator,* XGBoost *Extreme gradient boosting, *SVM *Support vector machine

**Table 3:** Area under the receiver operating characteristic curve (AUC) per vertebra with 95% confidence intervals in parentheses for different machine learning models

Vertebra	LASSO	Random Forest	XGBoost	SVM
L1	0.894 (0.750—1.000)	0.873 (0.768—0.979)	0.722 (0.399—1.000)	0.927 (0.847—1.000)
L2	0.836 (0.708—0.964)	0.785 (0.639—0.932)	0.889 (0.798—0.979)	0.884 (0.792—0.975)
L3	0.904 (0.813—0.996)	0.929 (0.852—1.000)	0.940 (0.858—1.000)	0.964 (0.920—1.000)
L4	0.790 (0.627—0.953)	0.743 (0.602—0.884)	0.811 (0.668—0.954)	0.773 (0.606—0.940)
L5	0.839 (0.736—0.943)	0.804 (0.685—0.923)	0.801 (0.683—0.918)	0.763 (0.636—0.890)
S1	0.793 (0.670—0.915)	0.745 (0.614—0.876)	0.779 (0.651—0.907)	0.789 (0.666—0.911)

*LASSO* Least absolute shrinkage and selection operator,* XGBoost *Extreme gradient boosting,* SVM *Support vector machine

**Table 4:** Diagnostic performance metrics and Brier scores of the different machine learning models

	Sensitivity (95% CI)	Specificity (95% CI)	Precision (95% CI)	F1 score (95% CI)	Brier score (95% CI)
LASSO	0.756 (0.662–0.846)	0.771 (0.721–0.819)	0.504 (0.414–0.589)	0.605 (0.521–0.679)	0.152 (0.131–0.176)
Random Forest	0.378 (0.275–0.482)	0.914 (0.878–0.945)	0.574 (0.442–0.704)	0.456 (0.346–0.553)	0.148 (0.124–0.176)
XGBoost	0.598 (0.494–0.702)	0.842 (0.798–0.883)	0.538 (0.438–0.638)	0.566 (0.481–0.650)	0.155 (0.127–0.185)
SVM	0.476 (0.365–0.585)	0.929 (0.896–0.958)	0.672 (0.547–0.789)	0.557 (0.452–0.650)	0.128 (0.108–0.151)

*CI* Confidence interval

### Feature importance

**Fig. 6:**
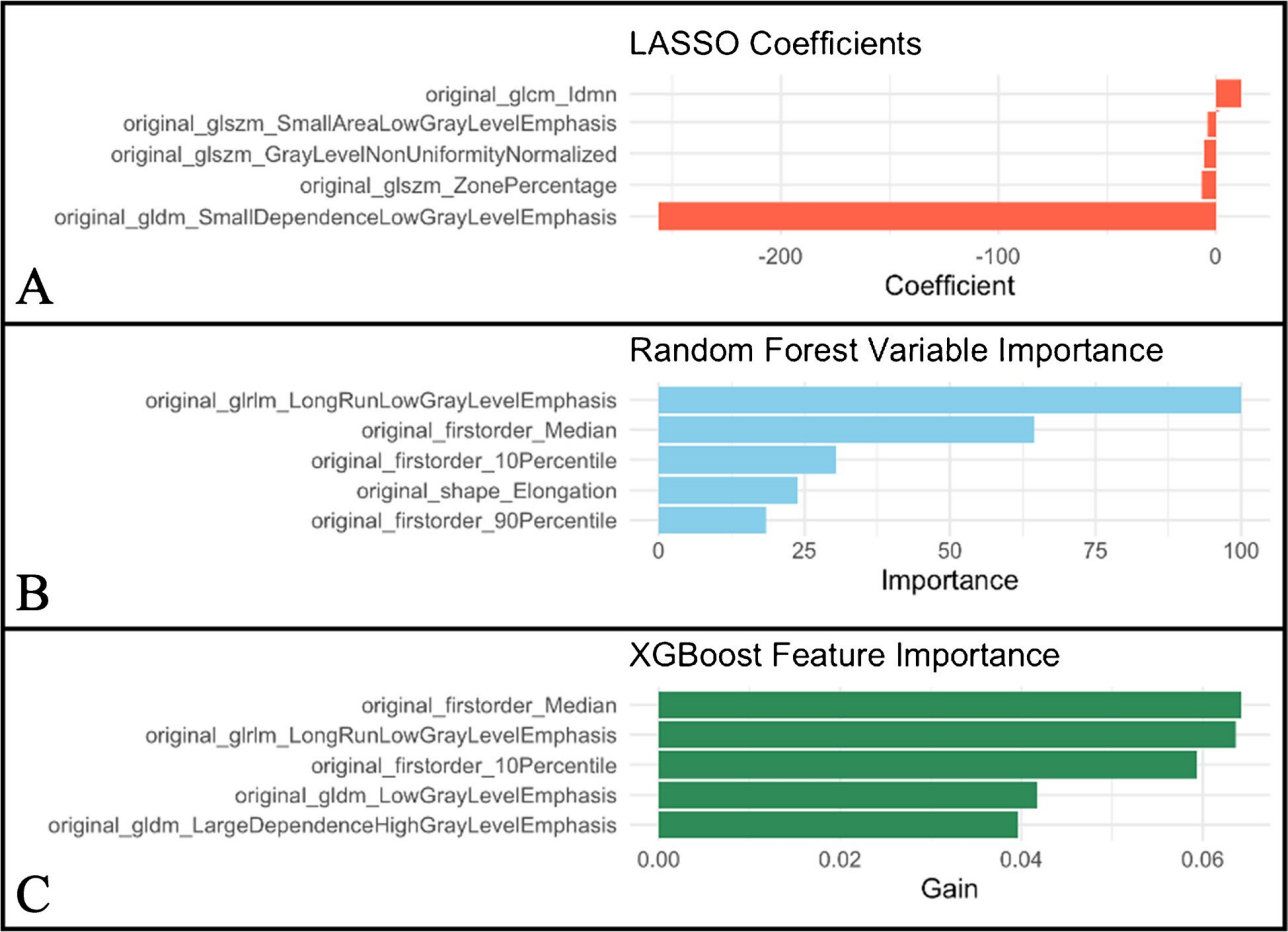
Feature importance across models. The most informative features across all models primarily reflect low-attenuation signal characteristics and textural heterogeneity within the vertebral body. These features capture the distribution and spatial organization of lower gray-level intensities. LASSO (**a**): Bars show non-zero coefficients from the final penalized logistic model at the optimal λ. Bar length reflects the absolute effect size on the log-odds scale; sign indicates direction of association (positive = higher predicted risk, negative = lower). Random Forest (**b**): Bars show relative variable importance rescaled by the forest’s impurity-based importance so the top feature = 100 and others are proportional. XGBoost (**c**): Bars show Gain, the average improvement in the loss function from splits using a feature; higher Gain indicates larger contribution to the model. *LASSO, Least Absolute Shrinkage and Selection Operator; XGBoost, Extreme Gradient Boosting*

The most important features across models were predominantly related to low-attenuation signal components and textural heterogeneity within the vertebral body. In particular, features emphasizing the presence and spatial distribution of lower gray-level intensities consistently ranked highest across models. While the specific feature rankings varied between algorithms, a common pattern was the importance of features reflecting heterogeneous low-density regions. Detailed feature importance rankings for each model are provided in Fig. 6.

## Discussion

This study compared different machine learning models for radiomics-based detection of Modic type 1 (MC-1) changes of the lumbar spine in PCD-CT images, using MRI as reference standard.

Radiomics features extracted from PCD-CT images demonstrated high discriminatory performance to detect MC-1 changes, which was comparable between the different machine learning models. Areas under the receiver operating characteristic curve (AUC) reached values of 0.81—0.84, along with acceptable calibration of probability estimates, which was lowest for Least Absolute Shrinkage and Selection Operator (LASSO) and Extreme Gradient Boosting (XGBoost).

To our knowledge, no prior machine learning-based studies have investigated the detection of Modic changes on CT. The results of our study provide novel insights by demonstrating that a texture-based radiomics approach can identify MC-1 changes, despite the very limited ability to detect these lesions on CT by visual assessment. In this context, the results of our study confirms that radiomics enables the extraction of quantitative imaging features that capture spatial heterogeneity beyond human visual perception. The most informative features were GLDM Small Dependence Low Gray Level Emphasis (LASSO), GLRLM Long Run Low Gray Level Emphasis (Random Forest), and first-order 10th percentile (XGBoost). These descriptors are intensity-sensitive, as they weight the lower-attenuation tail of the histogram (10th percentile) and the prevalence and spatial organization of low-gray-level voxels (low gray level emphasis). Importantly, these features capture heterogeneity and local patterns, unlike a region-of-interest-based approach, which only represents average Hounsfield units. Therefore, radiomics features may be useful especially in cases in which the visual attenuation of vertebrae with MC-1 changes is unremarkable on CT when assessed by the human observer. While the pathogenesis of MC-1 changes remains controversial [18], the texture changes found in our study can be explained by the histopathologic findings, which shows endplate disruption with ingrowth of fibrovascular granulation tissue and increased vascularity within adjacent marrow [18, 19].

The overall good discriminatory performance of our models aligns with a growing body of literature showing the usefulness of CT-based radiomics features across diverse pathologies of the spine. For tasks such as opportunistic osteoporosis screening on CT, differentiating acute versus chronic and benign from malignant vertebral compression fractures, reported CT-based radiomics AUCs span from 0.78 to 0.92 [20–22].

In order to improve model comparison, this study implemented a nested cross-validation strategy. For smaller datasets, this approach is considered preferable to single hold-out or non-nested k-fold schemes because it provides nearly unbiased performance estimates [23, 24]. This is achieved by evaluating on independent outer folds while confining feature selection, hyperparameter tuning, class weighting, and any probability calibration to the inner loop, thereby minimizing selection bias and data leakage. Given the ~ 23% prevalence of MC-1 changes among all vertebrae, inverse-frequency class weighting was applied during model training. This likely contributed to the observed acceptable model calibrations by reducing imbalance-induced bias in the predicted probabilities. However, the models were not specifically optimized for clinical decision thresholds, and no threshold-based decision analysis was performed.

From a clinical perspective, several potential implementation pathways for radiomics-based detection of MC-1 changes on CT can be considered. A realistic application would be opportunistic screening in patients undergoing CT for other indications, or in situations where MRI is contraindicated. Future studies including direct comparisons with radiologist assessment may help to further contextualize the added value of radiomics-based approaches over visual CT interpretation. However, several barriers to clinical implementation remain. External validation across institutions and scanner types is required to ensure generalizability, particularly given the dependence of radiomics features on acquisition and reconstruction parameters. Importantly, regulatory approval and prospective evaluation in real-world clinical settings are required before routine clinical use can be considered; however, most radiomics tools currently remain at a preclinical or research stage. Given the similar performance observed between the models, model choice may depend on whether sensitivity or specificity is prioritized.

The following limitations of this study should be acknowledged: First, this was a retrospective single-center analysis with a time interval between MRI and CT. Second, although a dedicated metal artifact reduction protocol was applied for all patients, residual artifacts may still have affected segmentation accuracy or radiomics feature extraction in a subset of cases. Third, we restricted our analysis to MC-1 changes and did not assess other Modic subtypes. This focus was deliberate, as MC-1 lesions are the subtype most consistently associated with symptomatic low back pain [4]. However, other Modic changes may have acted as potential confounders, as they might also influence CT-derived radiomics features in vertebrae which were classified as MC-1-negative. Fourth, multiple vertebrae per patient were analyzed, which may have influenced the precision of performance estimates by residual intra-patient correlation. This potential bias was mitigated by using patient-level cross-validation, which ensured that all vertebrae from a given patient were assigned exclusively to either training or test sets. Clustering was not explicitly modeled in the calculation of confidence intervals or AUC comparisons, which may have resulted in slightly optimistic estimates of statistical precision. Fifth, the spectral capabilities of PCD-CT, including virtual monoenergetic imaging, were not evaluated. Future studies may use these techniques, as they may improve radiomics feature stability, as previously demonstrated in a phantom study [13]. Sixth, external validation was not performed, which limits the generalizability of our findings. Last, including only patients with MC-1 changes might have introduced a selection bias that may limit the generalizability to unselected populations and influence performance metrics dependent on prevalence. However, these issues were addressed by performing the analysis at the vertebral level to include both affected and unaffected vertebrae and by applying class weighting during model training to account for class imbalance.

In conclusion, this study demonstrated that radiomics features derived from PCD-CT images enabled the detection of MC-1 endplate changes with good discriminatory performance across multiple machine learning models in an internally validated setting. The results demonstrate that CT-based radiomics can capture textural and intensity-based features of Modic changes that are not readily appreciable by visual inspection alone. Further external validation is required to assess generalizability and potential clinical utility of the observed findings.

## Data Availability

Data are available from the corresponding author upon reasonable request.
